# Radiology-Pathology Correlation of Peri-Anal Aggressive Angiomyxoma and Cellular Angiofibroma: Two Case Reports

**DOI:** 10.7759/cureus.70286

**Published:** 2024-09-26

**Authors:** Zainab Siddiqui, Shaikh Sayeed Iqbal, Ayoub Abdedzadeh, Paul Businge, Labib Al Ozaibi, Zainab Al Bloushi

**Affiliations:** 1 Radiology, Rashid Hospital, Dubai, ARE; 2 General Surgery, Rashid Hospital, Dubai, ARE

**Keywords:** aggressive angiomyxoma, angiomyxoid neoplasms, cellular angiofibroma, pelvic tumors, perianal tumors

## Abstract

Aggressive angiomyxoma and cellular angiofibroma are uncommon mesenchymal tumors. Imaging plays an important role in suggesting their diagnosis and in delineating the extent of the lesion. Additionally, histopathological examination provides the definite diagnosis.

An understanding of their histological features and correlating that with the key imaging features on radiology helps in narrowing the differential diagnosis, and thereby aids in management and outcome. We herein present a case of both entities in patients presenting with history of a palpable peri-anal mass. We describe the radiological and histological features of aggressive angiomyxoma and cellular angiofibroma and highlight the similarities and differentiating factors between them.

## Introduction

Aggressive angiomyxoma (AA) and cellular angiofibroma (CAF) are rare soft tissue tumors that arise from mesenchymal connective tissue. They are part of a unique subset of neoplasms that predominantly arise in the pelvis and share common histological features which include the presence of spindle to epithelioid stromal cells, an abundant matrix and prominent vessels [1]. The first description of aggressive angiomyxomas was reported in 1983 by Steeper and Rosai [2]. Following this, Fletcher et al. recognized a separate benign entity distinct from AA known as angiomyofibroblastoma (AMFB) [3]. Later, CAF was classified as a separate neoplasm by Nucci et al. in 1997 based on certain histological features [4]. 

Both tumors present with nonspecific clinical and physical examination findings, thus diagnosing and differentiating between them and similar histological variants poses a challenge for clinicians. They are frequently misdiagnosed as inguinal hernias, lipomas, Bartholin cysts, etc [5].

It is important to differentiate between AA and CAF owing to the differences in management and outcomes [1]. AAs are characterized by their tendency to locally insinuate adjacent structures and recur after excisions. This is in contrast to CAFs which so far have not been reported to show local recurrence [5,6].

Their final diagnosis is made on histopathology; however, diagnostic imaging plays a vital role in suggesting the diagnosis and in providing valuable anatomical details of these tumors therefore optimizing pre-operative planning. 

We herein present a case of CAF and AA and highlight the key imaging findings that aid in narrowing their diagnosis with important pathological correlation. Recognition of the imaging findings of these tumors is important so that they are considered in the differential diagnosis of peri-anal masses.

## Case presentation

Case 1

A 43-year-old female presented to the outpatient clinic with complaints of a perianal swelling that had been present for one month. The swelling was associated with intermittent dull pain in the perianal region. She had no significant past medical history or previous surgeries. Local examination revealed a soft, non-tender mass which was palpated in the peri-anal region at the 6 o'clock position, extending to the anal cleft. Vitals and laboratory tests were all unremarkable. 

**Figure 1:**
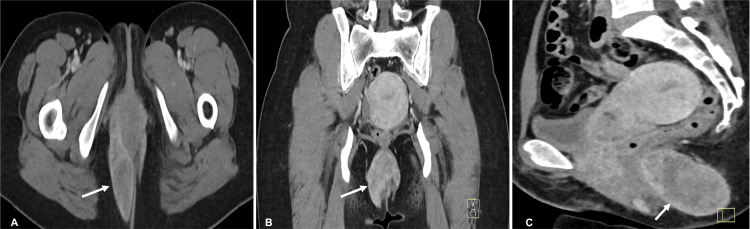
Contrast-enhanced CT scan of the pelvis. Axial (A), Coronal (B) and Sagittal (C) sections show a perianal solid mass lesion (blue arrow) extending across the anal sphincter showing heterogenous enhancement.

The patient subsequently underwent a contrast-enhanced pelvic CT scan for further evaluation. It revealed a large, well-defined, solid lesion in the peri-anal region extending in a horseshoe shape from the 6 o'clock to 2 o'clock position. It showed heterogeneous contrast enhancement and measured approximately 8.2 cm x 4.8 cm x 7 cm in dimensions (Figure 1). 

**Figure 2:**
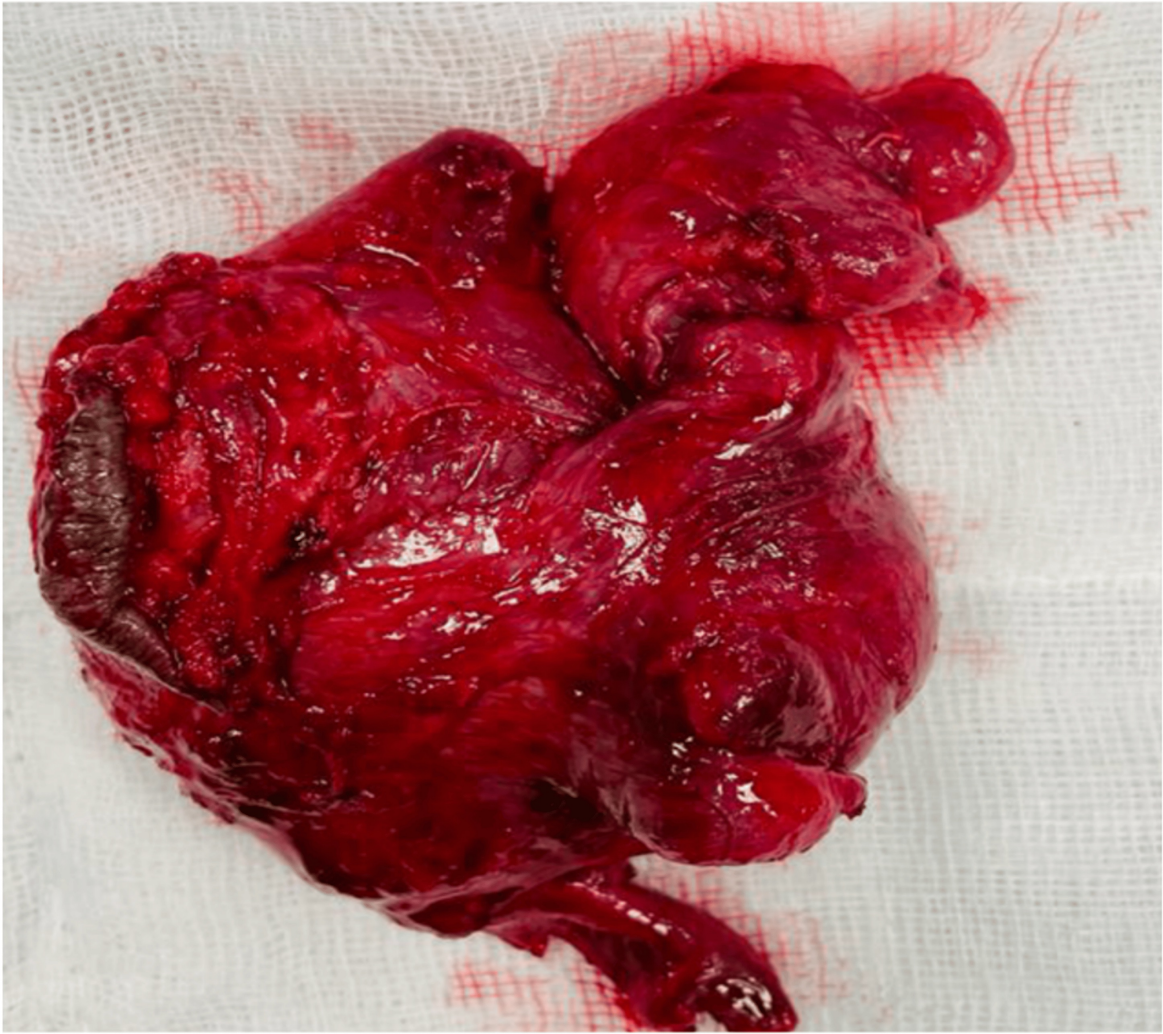
Gross pathological specimen of the resected tumor

**Figure 3:**
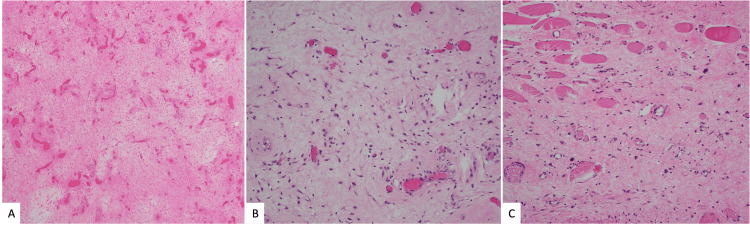
Histopathological examination of the tumor. Hematoxylin and Eosin-stained slides showing (A) Hypocellular myxoid stroma with multiple small and large blood vessels x 40 magnification. (B) Myxoid stroma with bland spindle stellate cells and thick hyalinized capillary vessels with extravasated RBCs x200 magnification. (C) Evidence of infiltrated atrophic skeletal muscle bundles x200 magnification.

She subsequently underwent surgical excision of the mass (Figure 2). Gross histological examination revealed a soft, rubbery, solid, homogeneous, gray-to-white spongy mass associated with polypoid projections. Microscopic hematoxylin and eosin staining demonstrated a hypocellular myxoid stroma with bland spindle stellate cells and thick hyalinized capillary vessels (Figure 3). Findings were consistent with AA. 

Case 2 

A 44-year-old female patient presented to the outpatient clinic with complaints of a large perianal swelling extending into the right gluteal region. The swelling was not associated with pain, discharge or palpable lymphadenopathy. Her past medical history was unremarkable and she had no history of previous surgeries. On clinical examination a firm 4 x 6 cm swelling was palpated in the right perianal region from 6 to 11 o'clock. Vitals and laboratory tests were all unremarkable. 

**Figure 4:**
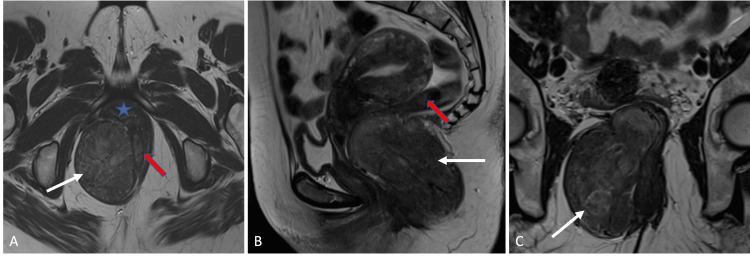
Axial (A) Sagittal (B) and Coronal (C) T2 weighted images show a well-defined mass (white arrow) demonstrating heterogenous predominantly low signal intensity. It is noted in the right ischiorectal fossa and demonstrates mass effect on distal rectum (red arrows) and vagina (star).

**Figure 5:**
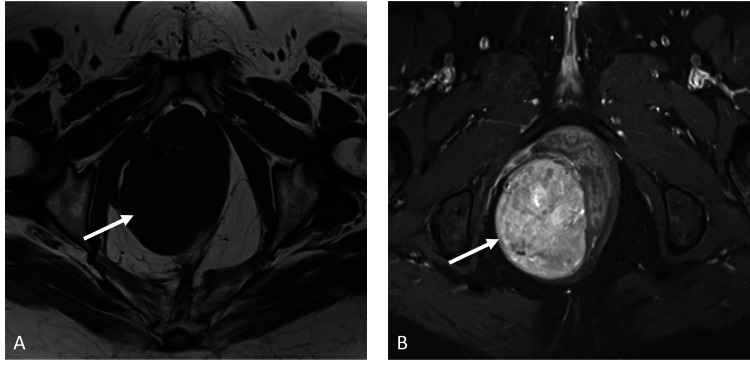
Axial T1 weighted image (A) and axial fat-suppressed T1 post-contrast image (B) shows a well-defined mass (white arrow) in the right ischiorectal fossa demonstrating low signal on T1 weighted image with avid enhancement on post-contrast image.

MRI revealed a well-defined, solid mass lesion in the right ischiorectal fossa measuring approximately around 5.5 cm x 5 cm x 10 cm in anterior-posterior, transverse and craniocaudal dimensions respectively. It elicited homogenous low signal intensity on T1-weighted images, heterogenous signal on T2-weighted images with intense heterogenous enhancement post contrast administration. The lesion demonstrated a compressive mass effect on the distal rectum, anal canal and vagina. Clear fat planes were appreciated between the lesion and adjacent structures with no evidence of infiltration. There was no evidence of restricted diffusion on imaging (Figures 4, 5). 

**Figure 6:**
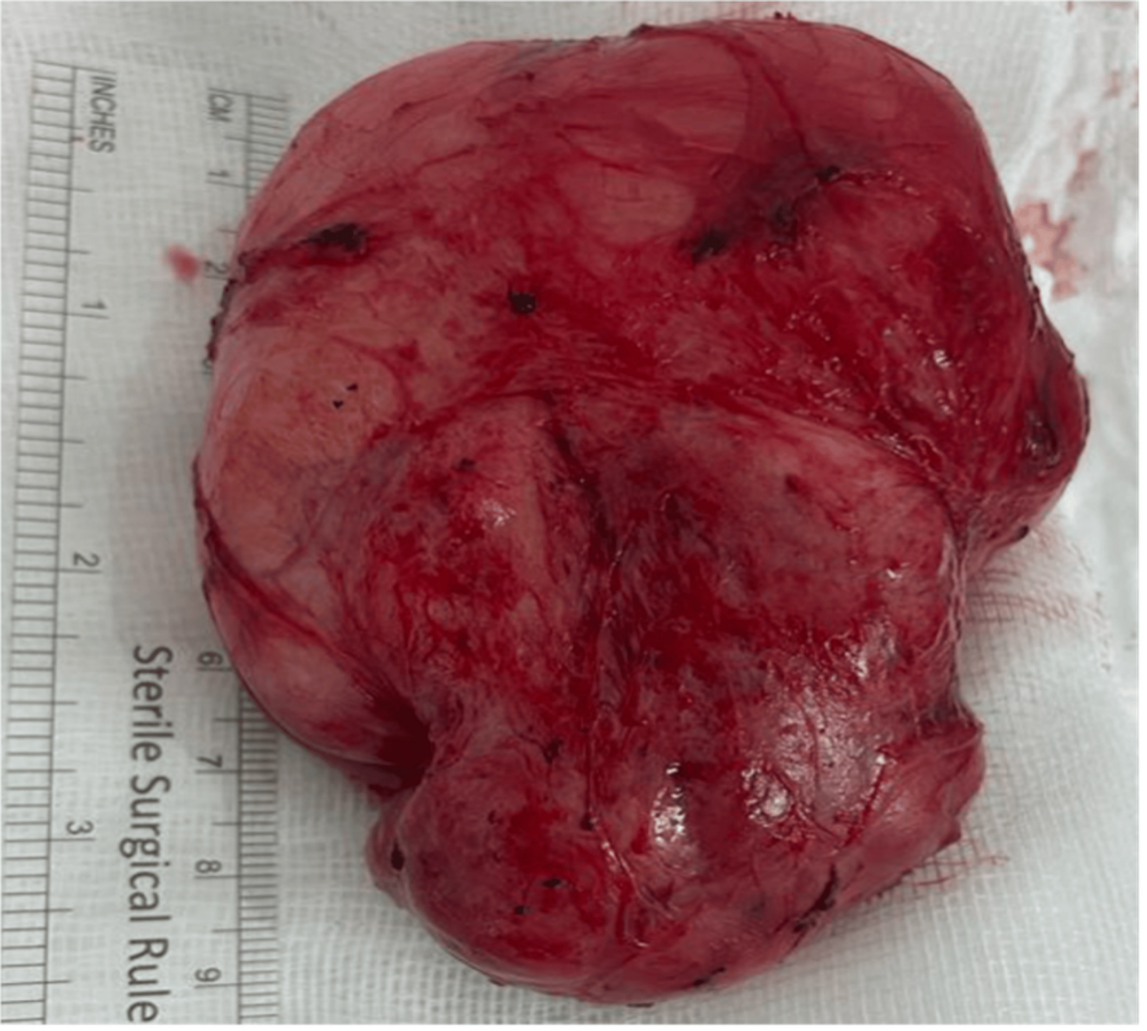
Gross pathological specimen of the resected tumor

**Figure 7:**
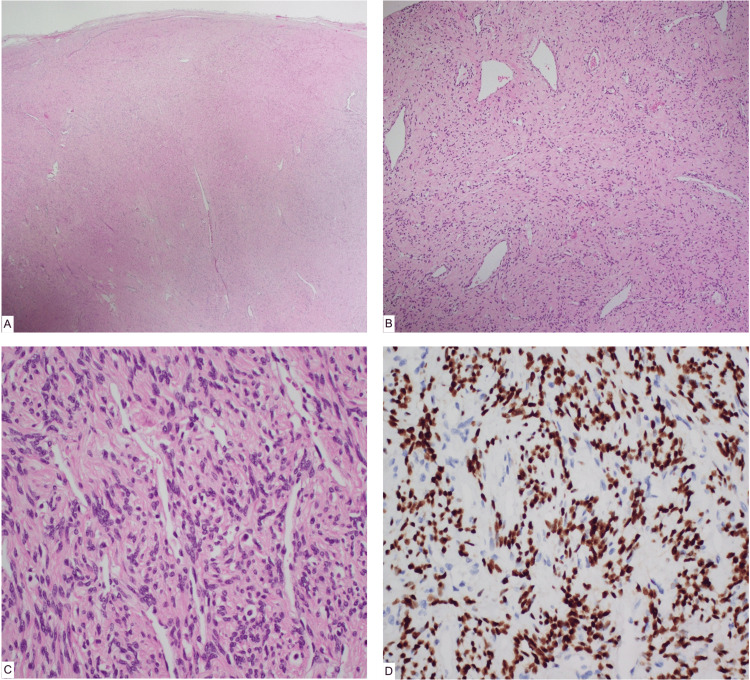
Histopathological examination of the tumor. (A) Hematoxylin and Eosin-stained slides showing (a) x20 magnification shows a well-defined almost capsulated stromal nodule. (B) x100 magnification shows spindle cell stroma with dilated thick staghorn-like blood vessels (C) x400 shows magnification cellular spindle cell proliferation with slit-like blood vessels. (D) Estrogen hormone receptor immunomarker positive nuclear staining of spindle cells.

Local excision of the mass was performed (Figure 6). Pathological examination showed a well-defined, circumscribed and almost capsulated mass nodule composed of spindle cells with variable cellularity, prominent vascularity, variable collagenous stroma and focal degenerative changes (oedema and hyalinization). A few hypocellular areas with myxedematous stroma were present. Some mitoses was seen (0 mitotic figures per 10 hpfs). There was no evidence of hemorrhage, significant atypia or necrosis. Prominent mast cells were present. The peripheral borders of the tumor appeared to be pushing and were not infiltrative. Immunohistochemistry was performed for the following markers: SMA, calponin, caldesmon, desmin, S-100, SOX10, CD34, CD117, estrogen and progesterone receptors. The neoplastic cells were strongly and diffusely positive for estrogen and progesterone receptors. The spindle cells were negative for CD34 and CD117, and the remaining tested markers were also negative. Ki67 showed a 2-3% proliferation index (Figure 7). These findings favored a diagnosis of CAF. 

## Discussion

We present two cases of rare mesenchymal tumors arising in the peri-anal region, AA and CAF. These are benign neoplasms categorized as angiomyxoid neoplasms, which most often arise in the perineal region; however, involvement of other anatomic sites has also been reported, such as the inguinal region, vulva, and genital tract; we currently report a case in the peri-anal region. These are slow-growing tumors that have the propensity to reach considerable sizes at the time of diagnosis [5-7].

Literature shows that AA occurs in a higher frequency in females with the median age of incidence around the fourth decade of life, whereas CAF has a relatively equal ratio of incidence between males and females, where the majority of cases occur in an older age group with the median age of incidence in the sixth decade of life [8].

Their diagnosis is a challenge clinically since these tumors present with vague symptoms and are frequently misdiagnosed as a bartholin cyst, lipoma, or a hernia, as the exact extent of these lesions may be undermined on physical examination. Upon presentation, patients may complain of a painless visible mass or experience symptoms of vulvovaginal pain, dysmenorrhea, or pressure symptoms from a mass effect upon adjacent structures [4,5,9]. 

AA primarily involves the deep soft tissues of the perineum and pelvis. It is characterized by the presence of disorganized blood vessels in an abundant myxoid stroma. The term ‘aggressive’ refers to its underlying nature to recur after surgical excision and its tendency to locally insinuate. A recurrence rate of up to 70% has been reported; hence, raising the suspicion on preoperative imaging is important to ensure complete surgical resection. Overall, however, they are considered benign tumors, with the incidence of metastasis being rare [10,11]. 

In comparison, CAF, also termed angiomyofibroma-like tumors, classically arises in the subcutaneous tissue, usually in the inguinoscrotal region for men and the vulvovaginal region for women. According to an analysis by Iwasa et al., these tumors are classically smaller in females with a median size of 2.8 cm. However, larger tumors, such as in our case (measuring 9 cm) have also been reported [12]. Additionally, their rate of recurrence is exceedingly lower [4,6]. 

The main radiological modalities that aid in the diagnosis of these tumors are CT and MRI, which are also essential for preoperative planning. Sonography, however, may be utilized in the initial assessment. 

AA demonstrates a homogenous and hypoechoic internal structure on ultrasound. Occasionally, they have also been described as frankly cystic masses. On CT imaging, AA shows a well-defined iso- to hypodense mass. The MRI features are reflective of the nature of the tumor itself. It demonstrates iso - hypointense signals on T1-weighted images when compared to muscles and iso- hyperintense signals on T2-weighted images. The high signal on T2-weighted images is attributed to the high water and myxoid contents of the tumor [13]. 

Additionally, the highly fibrovascular component of AA gives rise to a layered appearance on post-contrast imaging, also known as the ‘swirl sign’. Although this is not necessarily evident in all cases, Surabhi et al. demonstrated in a retrospective analysis that 83% of patients in the study revealed this appearance on MRI imaging, and 43% of patients revealed it on CT. A swirled appearance may also be seen on T2-weighted images without contrast administration [14]. Although MRI was not done in our patient, a heterogeneous, laminated enhancement was observed on CT in our case, and the diagnosis was suggested based on this. The tumor is commonly found in the perineal region; however, in our case, the mass was epicentered in the peri-anal region. When the tumors attain considerable sizes, displacement, rather than invasion, of adjacent structures is commonly seen [13]. 

On imaging, CAF is characteristically a well-circumscribed tumor that demonstrates heterogeneous enhancement on CT post intravenous contrast administration [7]. On MR imaging, like AA, CAF appears iso-intense to the muscles on T1-weighted images. The T2 signal intensities are variable depending on the content of collagenous stroma, myxoid matrix, and fat tissue. As a result, the signal intensity may range from hypo to hyperintense. A hypointense signal usually reflects a high amount of collagen tissue within the lesion. Post-contrast imaging typically demonstrates avid and intense solid enhancement. Unlike aggressive angiomyxomas, cellular angiofibromas do not tend to locally infiltrate and have an overall low rate of recurrence rate [6,8].

The imaging features of AA and CAF significantly overlap. However, distinguishing features include that CAF frequently exhibits avid, homogenous enhancement on post-contrast MRI sequences, while a laminated or swirling enhancement is preferentially seen with AA [8].

On pathological analysis of AA, gross examination typically reveals a soft, well-defined, gelatinous, and polypoid mass, with finger-like projections ranging in size from a few centimeters to 20 cm or even more, as in our case. Microscopically, these tumors are composed of loosely arranged, spindle- to stellate-shaped cells with thick-walled vessels of various calibers embedded in a myxoid matrix. Cellular atypia or mitotic activity has been shown to be rare. Regarding immunohistochemistry, AA lacks a definitive marker profile that would be diagnostic. However, tumor cells often show positivity for vimentin, desmin, smooth muscle actin, CD34 or estrogen, and progesterone receptors [1].

Conversely, CAF may share some overlapping histological features with AA. Characteristically, these tumors are composed of bland spindle cells arranged in short intersecting fascicles. Unlike AA, the stroma in CAF is more collagenous, characterized by short, wispy collagen bundles with abundant medium-sized vessels that have a hyalinized wall. Adipose tissue may also be present in some tumors (typically around the periphery of the tumor), and sarcomatous transformation has been reported. Immunohistochemical staining for CD34 and vimentin is usually positive in CAF, while the expression of estrogen and progesterone receptors is variable [15,16]. 

Given the overlap in immunoreactivity between both tumors, it is important to highlight that immunohistochemistry alone cannot reliably differentiate between AA and CAF. However, it has been reported that CAF stains poorly for desmin and alpha-smooth muscle actin, whereas AA more commonly stains positive for them [17]. 

The definitive management for both tumors is surgical excision [11]. Given the nature of AA to locally recur, a goal of achieving complete resection with tumor-free margins is often emphasized to decrease the risk of recurrence [11]. This, however, may not be feasible in all cases where radical surgery to achieve negative margins may lead to increased operative morbidity. Therefore, the benefits and risks of the extent of surgical excision should be carefully evaluated [5]. On the other hand, the need for wide excision has been questioned in the surgical excision of CAF given its low rate of recurrence even in cases with positive margins [4]. As a result, it is important to try to suggest a diagnosis on preoperative imaging due to the impact on operative planning. Imaging delineates the tumoral extent and determines the optimal surgical approach for achieving tumor-free margins as much as possible, a factor of importance particularly in management of AA. It is also utilized post-operatively for follow-up of patients for any signs of recurrences. 

Furthermore, apart from surgical management, medical treatment with hormonal therapy has been reported for AA using gonadotropin-releasing hormone (GnRH) agonists or tamoxifen in view of their estrogen and progesterone receptor positivity. GnRH agonists may be considered to reduce the size of the tumor preoperatively or as adjuvant therapy to prevent recurrence post-excision in the management of AA [17]. Imaging following hormonal treatment of the tumors can demonstrate a decrease in size, as well as decreased signal intensity on T2-weighted sequences and decreased enhancement [8]. Conversely, the use of hormonal therapy has not been explored as much in the treatment of CAF. 

## Conclusions

In conclusion, AA and CAF are uncommon mesenchymal tumors, which should be considered in the differential diagnosis of solid tumors in the peri-anal region. Differentiating factors between both tumors include that AA more commonly occurs in women, is larger in size, typically originates in the deep pelvis, and has a higher rate of recurrence post-operatively. CAF, on the other hand, frequently occurs in the superficial soft tissues, typically in the vulvovaginal region or inguinoscrotal region, and tends to be smaller in size.

Microscopically, AA is distinguished from CAF by its hypocellularity, consisting of scant spindle-shaped cells in a myxoid stroma, whereas CAF is composed of spindle-shaped cells present in a predominant fibrous stroma, containing wispy collagen bundles and numerous hyalinized small-medium blood vessels. These histological findings are also reflected in the imaging findings of AA and CAF, which serve as discriminating features on imaging. AA demonstrates a swirled appearance typically on post-contrast MRI sequences and T2-weighted sequences due to its underlying myxoid content, and CAF shows avid, solid enhancement post-contrast on both MRI and CT, reflecting its underlying nature of a vascular-rich fibroblastic neoplasm. 

Overall, imaging plays an important role in characterizing these tumors and aids in surgical planning preoperatively. Their final diagnosis is made on histopathology. 

## References

[1] Haroon S, Irshad L, Zia S (2022). Aggressive angiomyxoma, angiomyofibroblastoma, and cellular angiofibroma of the lower female genital tract: related entities with different outcomes. Cureus.

[2] Steeper TA, Rosai J (1983). Aggressive angiomyxoma of the female pelvis and perineum. Report of nine cases of a distinctive type of gynecologic soft-tissue neoplasm. Am J Surg Pathol.

[3] Fletcher CD, Tsang WY, Fisher C, Lee KC, Chan JK (1992). Angiomyofibroblastoma of the vulva. A benign neoplasm distinct from aggressive angiomyxoma. Am J Surg Pathol.

[4] Mandato VD, Santagni S, Cavazza A, Aguzzoli L, Abrate M, La Sala GB (2015). Cellular angiofibroma in women: a review of the literature. Diagn Pathol.

[5] Dahiya K, Jain S, Duhan N, Nanda S, Kundu P (2011). Aggressive angiomyxoma of vulva and vagina: a series of three cases and review of literature. Arch Gynecol Obstet.

[6] Koo PJ, Goykhman I, Lembert L, Nunes LW (2009). MRI features of cellular angiomyofibroma with pathologic correlation. J Magn Reson Imaging.

[7] Kamitani R, Matsumoto K, Fujiwara S (2020). A case of inguinal cellular angiofibroma. IJU Case Rep.

[8] Figueiredo G, O'Shea A, Neville GM, Lee SI (2022). Rare mesenchymal tumors of the pelvis: imaging and pathologic correlation. Radiographics.

[9] Bensalah A, Charifi Y, Ousrouti LT (2021). Perineal and pelvic aggressive angiomyxoma: imaging finding in an uncommon case report. Radiol Case Rep.

[10] Kiran G, Yancar S, Sayar H, Kiran H, Coskun A, Arikan DC (2013). Late recurrence of aggressive angiomyxoma of the vulva. J Low Genit Tract Dis.

[11] Chan YM, Hon E, Ngai SW (2000). Aggressive angiomyxoma in females: is radical resection the only option?. Acta Obstet Gynecol Scand.

[12] Iwasa Y, Fletcher CD (2004). Cellular angiofibroma: clinicopathologic and immunohistochemical analysis of 51 cases. Am J Surg Pathol.

[13] Outwater EK, Marchetto BE, Wagner BJ, Siegelman ES (1999). Aggressive angiomyxoma: findings on CT and MR imaging. AJR Am J Roentgenol.

[14] Surabhi VR, Garg N, Frumovitz M, Bhosale P, Prasad SR, Meis JM (2014). Aggressive angiomyxomas: a comprehensive imaging review with clinical and histopathologic correlation. AJR Am J Roentgenol.

[15] Schoolmeester JK, Fritchie KJ (2015). Genital soft tissue tumors. J Cutan Pathol.

[16] Chen E, Fletcher CD (2010). Cellular angiofibroma with atypia or sarcomatous transformation: clinicopathologic analysis of 13 cases. Am J Surg Pathol.

[17] Maciejczyk A, Bartecki K, Czarnecka A (2024). Diagnostics and treatment of aggressive angiomyxoma. Oncol Clin Pract.

